# Understanding caregiver descriptions of initial signs and symptoms to improve diagnosis of metachromatic leukodystrophy

**DOI:** 10.1186/s13023-022-02518-z

**Published:** 2022-10-04

**Authors:** F. Eichler, Caroline Sevin, M. Barth, F. Pang, K. Howie, M. Walz, A. Wilds, C. Calcagni, C. Chanson, L. Campbell

**Affiliations:** 1 Center for Rare Neurological Diseases, Massachusetts General Hospital, Boston, MA USA; 2 Harvard Medical School, Boston, MA USA; 3 Service de Neuropédiatrie, centre de reference des leucodystrophies et leucoencephalopathies genetiques de cause rare, CHU Paris-Sud-Hôpital de Bicêtre, Le Kremlin-Bicêtre, France; 4 Service de Génétique, Hôpital Universitaire d’Angers, Angers, France; 5 Orchard Therapeutics, 245 Hammersmith Road, London, W6 8PW UK; 6 Magnolia Innovation, Hoboken, NJ USA

**Keywords:** Metachromatic leukodystrophy, MLD, Initial symptoms, Disease onset, Diagnostic delay, Caregiver experience, Caregiver language, Early-onset, Late infantile, Juvenile

## Abstract

**Background:**

Metachromatic leukodystrophy (MLD), a relentlessly progressive and ultimately fatal condition, is a rare autosomal recessive lysosomal storage disorder caused by a deficiency of the enzyme arylsulfatase A (ARSA). Historically management has been palliative or supportive care. Hematopoietic stem cell transplantation is poorly effective in early-onset MLD and benefit in late-onset MLD remains controversial. Hematopoietic stem cell gene therapy, Libmeldy (atidarsagene autotemcel), was recently approved by the European Medicines Agency for early-onset MLD. Treatment benefit is mainly observed at an early disease stage, indicating the need for early diagnosis and intervention. This study contributes insights into the caregiver language used to describe initial MLD symptomatology, and thereby aims to improve communication between clinicians and families impacted by this condition and promote a faster path to diagnosis.

**Results:**

Data was collected through a moderator-assisted online 60-min survey and 30-min semi-structured follow-up telephone interview with 31 MLD caregivers in the United States (n = 10), France (n = 10), the United Kingdom (n = 5), and Germany (n = 6). All respondents were primary caregivers of a person with late infantile (n = 20), juvenile (n = 11) or borderline late infantile/juvenile (n = 1) MLD (one caregiver reported for 2 children leading to a sample of 32 individuals with MLD). Caregivers were asked questions related to their child’s initial signs and symptoms, time to diagnosis and interactions with healthcare providers. These results highlight the caregiver language used to describe the most common initial symptoms of MLD and provide added context to help elevate the index of suspicion of disease. Distinctions between caregiver descriptions of late infantile and juvenile MLD in symptom onset and disease course were also identified.

**Conclusions:**

This study captures the caregiver description of the physical, behavioral, and cognitive signs of MLD prior to diagnosis. The understanding of the caregiver language at symptom onset sheds light on a critical window of often missed opportunity for earlier diagnosis and therapeutic intervention in MLD.

**Supplementary Information:**

The online version contains supplementary material available at 10.1186/s13023-022-02518-z.

## Introduction

### Background and epidemiology

Metachromatic leukodystrophy (MLD) is a rare autosomal recessive lysosomal storage disorder caused by mutations in the *ARSA* gene leading to a deficiency of the enzyme arylsulfatase A (ARSA) [1]. The decreased activity of ARSA results in the accumulation of sulfatides in the central and peripheral nervous systems, leading to microglial damage, neurodegeneration, progressive demyelination, and loss of motor and neurocognitive function [1–4]. MLD is characterized by progressive motor and cognitive deterioration [1, 5]. Typically, MLD is classified into 3 subtypes by age of onset of first symptoms, including late infantile (symptom onset ≤ 30 months of age); juvenile, subdivided into early juvenile (onset > 30 months and < 7 years) and late-juvenile (onset ≥ 7 years and < 17 years); and adult (onset ≥ 17 years). Late infantile and early juvenile MLD together (onset < 7 years) is more broadly classified as “early-onset MLD” (symptoms onset < 7 years of age) [3, 6, 7]. Worldwide MLD prevalence is 1 in 40,000 to 160,000 with 50–60% of patients found with the late infantile form, 20–30% diagnosed with juvenile form, and 15–20% of patients diagnosed with the adult form [8, 9].

### Diagnosis

Upon suspicion of disease, genetic analysis for *ARSA* and *PSAP* mutations, brain imaging, and biochemical testing of ARSA enzymatic activity are used in the diagnosis of MLD. MRI is typically the first step in directing clinicians to conduct biochemical and genetic testing, which can ultimately lead to a definitive diagnosis [10]. Specific characteristics of late infantile MLD include abnormalities of nerve conduction and demyelinating neuropathy, which can be detected with magnetic resonance imaging. In juvenile MLD, initial indicators of MLD revealed through imaging include central and periventricular white matter changes. While MLD can be detected in fetus and newborn genetic screening, the condition is not typically included in these types of early testing due to its rarity [9]. Therefore, these clinical tests are not initiated until MLD is suspected, which can lead to significant delays in biochemical testing due to the often non-specific nature of initial signs of MLD [1].

### Treatment

Historical management has been palliative or supportive care. Limited benefit has been observed in the use of hematopoietic stem cell transplantation (HSCT) [5, 11]. Transplant benefit is only observed in individuals whose disease has not significantly progressed, indicating a need for early diagnosis and intervention [12]. Transplantation after the presentation of motor and/or cognitive symptoms significantly limits the chance of the procedure’s success and can often result in rapid decline post-transplant [11]. Gene therapy and enzyme replacement therapy are being investigated for the treatment of MLD [13]. Ex vivo hematopoietic stem cell gene therapy, Libmeldy (atidarsagene autotemcel) was recently approved by the European Medicines Agency [14].

### Research focus

Early recognition of MLD is crucial to increase the chance of individuals with MLD qualifying for and benefiting from therapy. However, the initial signs and symptoms can be subtle and non-specific, and can go unrecognized for months and years [1]. From a caregiver perspective, this time between symptom onset and diagnosis is characterized by uncertainty, frustration, and fear. While there are a few studies focused on MLD that highlight or include a caregiver perspective, there is continued need to better understand, via caregiver language and descriptions, what patients’ experiences look like early in the disease process to enhance our understanding of how to identify MLD more quickly [15, 16].

The goal of this paper is to offer insight into the caregiver descriptions of the initial symptomatology of MLD and ultimately to improve communication between clinicians and families impacted by this condition. This paper characterizes the initial symptoms in the words of caregivers, perceived speed of disease progression, and time to diagnosis. Distinctions between late infantile and juvenile MLD in symptom onset and disease course were also identified. The results from this study will augment findings from previous publications in order to provide further clarity and enhance the diagnostic odyssey.

## Methods

Data were collected through an online survey and qualitative interviews with MLD caregivers in the United States (US), France (FR), Germany (DE), and the United Kingdom (UK). The Research Project was carried out in part on the Leuconnect platform in partnership with ELA International (ELA 2019-P003). Data were collected as part of a larger research study on disease burden and quality of life as reported by caregivers of children diagnosed with MLD. The study was managed by Magnolia Innovation on behalf of the study sponsor, Orchard Therapeutics.

### Study design and data collection

Study protocol and all study material were approved by an external IRB (provider: Pearl IRB, Indianapolis, IN). The survey instrument was designed by Magnolia Innovation and reviewed by clinicians with expert knowledge of MLD, and representatives of patient advocacy groups. Data was collected through moderator-assisted online surveys (length: 60-min) and semi-structured telephone interviews (length: 30-min). In the survey, caregivers were asked to describe initial symptoms (“in hindsight, what were the first clues that all was not well with your child?”), confirm if and when they sought medical advice after noticing these clues (“did you seek medical advice from a doctor after noticing these first clues?”; “what symptoms ultimately made you seek out medical advice?”), how much time passed until they sought said advice (“how much time passed from when you saw these first clues to when you sought medical advice?), at what age they noticed development regression (“at what age did you notice your child’s developmental regression?”), and at what age their child was diagnosed (“at what age was your child diagnosed?”). Other topics were included in this survey as part of a broader study on caregiver burden, which were not included in this paper but can be referenced in companion paper, *An International Study of Caregiver-Reported Burden and Quality of Life in Metachromatic Leukodystrophy* [17]. During the follow-up interviews, respondents were asked to provide additional details around their interactions with healthcare providers and challenges experienced leading up to diagnosis.

### Sample/respondent selection

Respondents were contacted and recruited with the help of patient advocacy groups. Patient advocacy groups’ members were notified of the opportunity to participate in this study and instructed to connect directly with Magnolia Innovation to confirm eligibility. The sponsor of the study had no direct contact with interested participants. During the recruitment process respondents were briefed on study objectives and methodology, answered initial screening questions, provided written consent to participate, and confirmed the MLD diagnosis. Surveys were completed by 31 respondents from four countries (US = 10, FR = 10, DE = 6, UK = 5). Twenty-nine of the 31 respondents participated in follow-up interviews (2 declined to participate in the follow-up conversation) and three survey respondents were also joined by their spouse/partner. A total of 32 individuals with MLD were included in the analysis (US = 10, FR = 11, DE = 6, UK = 5)—one caregiver provided information on two children with MLD while all others shared information on one child with MLD. Survey calls and interviews were conducted in English (for US and UK), German, and French.

All respondents were asked to confirm their comfort level with speaking about their experiences and were provided the opportunity to opt out at any point in the study. Special precautions were taken to adjust questions appropriately for caregivers of children who had passed away. Respondents were directed to available mental health support services where relevant. No children of caregivers passed away during the study period, and therefore no acute interviewee psychological support was required.

### Data management/analysis

Survey data and interview transcripts were further anonymized to blind the sponsor to respondent identity. Survey answers on initial symptoms were coded to identify what type of symptoms were most commonly observed at disease onset. Frequency of codes are reported for total sample and by onset types, for late infantile onset and juvenile onset. Given the small sample size, this study provides descriptive statistics.

## Results

### Respondent characteristics

#### Caregiver background

In total, 31 caregivers were included in this study, all of whom were parents of children with MLD. Twenty-six of the 31 caregivers interviewed were mothers (83.9%), and the remaining caregivers were fathers (one stepfather) of the children with MLD (5/31; 16.1%). All but four children with MLD were alive at the time of research (28/32; 87.5%).

#### Symptom onset type breakout

Individuals were categorized as late infantile MLD if their symptom onset began at, or prior to, 30 months of age. Juvenile MLD categorization was defined by symptom onset between the ages of 31 months and 17 years of age. Twenty individuals (62.5%) were reported to have late infantile onset and 11 individuals (34.4%) were reported as juvenile onset. One caregiver (3.1%) reported their child received a diagnosis of borderline late infantile/juvenile—this respondent’s data was included in qualitative findings, but was excluded from any onset type categorization or statistical analysis. The mean age at the time of interviews of these late infantile and juvenile MLD individuals was 5.2 years and 15.6 years, respectively (four individuals had passed away prior to interview and their ages are not represented here; the late infantile individuals [n = 3] had died at 3.5, 4, and 7.7 years old; the juvenile individual [n = 1] had died at 32 years old) (Table 1).

**Table 1:** MLD onset type by country

Onset type	MLD individuals, n (%)				
	Overall	US, n (%)	FR, n (%)	DE, n (%)	UK, n (%)
Total individuals	32 (100)*	10 (31.3)	11 (34.4)	6 (18.8)	5 (15.6)
Late infantile onset (age range ≤ 30 months)	20 (62.5)	6 (60.0)	7 (63.6)	4 (66.7)	3 (60.0)
Juvenile onset					
All juvenile (age range > 30 months to < 17 years)	11 (34.4)	3 (30.0)	4 (36.4)	2 (33.3)	2 (40.0)
Early (age range > 30 months to < 7 years)	8 (25.0)	2 (20.0)	3 (27.3)	1 (16.7)	2 (40.0)
Late (age range 7 to < 17 years)	3 (9.4)	1 (10.0)	1 (9.1)	1 (16.7)	0 (0.0)

*Borderline late infantile/juvenile onset case not included in breakout of US sample onset types

#### Sample diagnosis and treatment

All MLD individuals but two, who were diagnosed through genetic testing as a result of a sibling’s diagnosis, were diagnosed symptomatically. Of those alive at the time of interview, all but two were at advanced stages of their disease (i.e., severe cognitive impairment and loss of trunk control). On average, time between onset of the MLD individual’s symptoms and caregiver participation in interviews was 5.7 years (range 1–16.3 years; 3.8 years for caregivers of those diagnosed with late infantile MLD and 8.2 years for juvenile). Five individuals in this study received HSCT (15.6%) (Table 2).

**Table 2:** Demographics of MLD individuals as reported by their respective caregiver respondent

Characteristics	Overall (n = 32*)	Late infantile (n = 20)	Juvenile (n = 11)
Sex			
Female, n (%)	21 (65.6)	12 (60.0)	9 (81.8)
Male, n (%)	11 (34.4)	8 (40.0)	2 (18.2)
Current age (years)^+^			
Mean	9.2	5.2	15.6
Median	7.4	4.5	14.3
Range	2.3–30.3	2.3–11.1	8.0–30.3
Time elapsed since symptom onset (years)^+^			
Mean	5.7	3.8	8.2
Median	4.8	3.0	7.5
Range	1.0–16.3	1.0–9.6	2.0–16.3

*One respondent is not accounted for in onset type breakout due to reportedly being a borderline late infantile/juvenile case

^+^Excludes 4 individuals (12.5%) who were not alive at the time of interview

### Caregiver language used to describe initial signs and symptoms of MLD

#### Caregiver descriptions of early MLD signs and symptoms

Specific terminology, or language, used by caregivers was captured in detail to see how the early signs of MLD are identified and described by caregivers (Table 3). Caregivers used a variety of terms to describe what are presumably some of the typical early symptoms. For example, caregivers described what is likely clonus as “twitching”, “shaking”, or “stroke-like movements”. To describe difficulty walking, some used more clinical terms, such as “gait spasticity” while others were more general in their descriptions (e.g., “unstable walking”).

#### Observed changes in daily activities

Observed changes in behavior or personality, such as sudden stubbornness or loss of interest in activities, were cited by 6 caregivers (18.8%). One caregiver articulated the changes they noticed in their child with juvenile MLD by recollecting her new difficulties in running errands and changes in behavior, including enuresis (see Additional file 1: “Observed Changes in Daily Activities” for quotes from caregivers).

#### Peer comparisons

Caregivers used peer comparisons to explain the apparent gaps or delays in meeting age-related milestones. As one caregiver articulated, their child with late infantile onset MLD was “slower than the average child that we could see” in terms of meeting developmental milestones (see Additional file 1: “Peer Comparisons” for quote from a caregiver).

#### Describing developmental red flags in caregiver language

There was also limited direct use of the words “developmental delays, stagnation, or regression”. Instead, caregivers shared examples of activities that their child was delayed in or unable to achieve, such as “could never run freely”. Indications of developmental delays were phrased as milestones that they felt their child was “late” to accomplish such as, “started walking late as a baby” or “late to stand up”. Signs of developmental stagnation were described as milestones the child was never able to reach—such as “never able to ride a bike” or “could never walk freely”. Concerns of developmental regression were described in terms of the lost capabilities of their child, e.g., excelling in math but then becoming unable to add (Table 3).

**Table 3:** Language used by caregivers to describe initial signs and symptoms of MLD

SECTION I: Language used by caregivers to describe initial signs and symptoms of MLD		
Symptom category	Language used by caregivers	
Coordination difficulties	Abnormal gait Broad-based gait Delayed walking, difficult walking, strange posture Delayed when walking, many falls, gait sluggish Early to crawl, but late to stand up and hold onto things Has never been able to walk freely, twisted foot while walking Loss of balance, tripping Never walked, left was weaker than her right side Not progressing with walking (started taking first steps but did not progress after) Pain when walking, motor problems indicated by the teacher	Problems with motor development Slow motor skills Stagnation of motor development Started to lose balance Struggled to run- uncoordinated, clumsy, started walking late as a baby Trouble walking Unstable sitting, walking Unstable walking, never able to ride a bike Unsteady gait Wasn’t getting on the couch anymore Wasn’t walking
Clonus/tremor	Arm movement as if after a Stroke Clonus Developed a small tremor in Hands Hand tremors Shake really bad after naps, Foot tremoring	slight tremor slight tremor, eye lid twitching (that pediatrician noticed on regular checkup) tremors very mid absences
Comprehension challenges	Ability to do math, top of his class in 1st grade, 2nd grade couldn't monitor progress and didn't know he was struggling, 3rd grade couldn't add Appearing sleepy and dazed Cognitive delays Difficulty learning (learning vocabulary)	Forgetful- getting lost Gaps between achieving milestones was getting bigger Lack of concentration, issues with concentration levels Only 6 words Regression in writing
Changes in personality/behavior	Behavioral disorders Much crying Obstinate Peeing pants in school Personality changes, impulsive behavior, issues with sleep, loss of interest in activities that […] used to be interested in	Severe fatigue, nocturnal awakenings
Vision issues	Strabismus, nystagmus Sudden squint	Went cross-eyed overnight

SECTION II: Language used by caregivers to describe specific developmental issues of MLD		
Developmental issues	Language used by caregivers	
Developmental delays *Reaching milestones slowed*	Delayed walking Developmental delays, small, cognitive delays Early to crawl, but late to stand up and hold onto things, gaps between milestones was getting bigger	Late walker Difficulty learning (learning vocabulary) Slower than average child, wasn’t walking Started walking late as a baby
Developmental stagnation *Milestones never met*	Could never walk freely Could never walk independently Development not progressing Motor development stagnation	Never able to ride a bike Never walked Not progressing with walking (started taking first steps but did not progress after) Stagnation of motor development
Developmental regression *Losing milestones that were previously reached*	Ability to do math, top of his class in 1st grade, 2nd grade couldn't monitor progress and didn't know he was struggling, 3rd grade couldn't add Appearing sleepy and dazed, loss of interest in activities that he used to be interested in Issues with concentration levels, little bit of regression (not age-appropriate behavior)	Forgetful- getting lost, peeing in pants at school Loss of balance Only 6 words, regressing Regression in writing Trouble walking (later on) Unsteady gait, sudden squint Wasn’t getting on the couch anymore Went cross-eyed overnight and started to lose her balance

#### Late infantile versus juvenile

Differences in age of onset of symptoms and in turn, differences in relevant developmental milestones can impact the types of symptoms described by caregivers. The ability to walk was commonly identified by caregivers of children with late infantile MLD as an expected milestone that their child either struggled with or was never able to achieve. Other physical observations were commonly mentioned amongst caregivers of children with late infantile MLD such as signs of clonus and vision issues. Conversely, in juvenile patients, caregivers’ observations were often descriptive of the specific cognitive and behavioral changes noticed in their children (Table 4).

**Table 4:** Language used by caregivers of children with late infantile versus juvenile MLD to describe specific developmental issues of MLD

Symptom category	Late infantile (n = 20)	Juvenile (n = 11)
Coordination difficulties	Delayed walking, difficult walking, strange posture Delayed when walking, many falls, gait sluggish Early to crawl, but late to stand up and hold onto things Has never been able to walk freely, twisted foot while walking Never walked, left was weaker than her right side Not progressing with walking (started taking first steps but did not progress after) Problems with motor development Slow motor skills Stagnation of motor development Started to lose balance Struggled to run- uncoordinated, clumsy, started walking late as a baby Trouble walking Unstable sitting, walking Unsteady gait Wasn’t getting on the couch anymore Wasn’t walking	Abnormal gait Broad-based gait Loss of balance, tripping Pain when walking, motor problems indicated by the teacher Unstable walking, never able to ride a bike
Clonus/tremor	Clonus Developed a small tremor in hands Shake really bad after naps, foot tremoring Slight tremor Slight tremor, eye lid twitching (that pediatrician noticed on regular checkup) Tremors Very mid absences	Arm movement as if after a stroke Hand tremors
Comprehension challenges	Appearing sleepy and dazed Cognitive delays Gaps between achieving milestones was getting bigger Only 6 words	Ability to do math, top of his class in 1st grade, 2nd grade couldn't monitor progress and didn't know he was struggling, 3rd grade couldn't add Difficulty learning (learning vocabulary) Forgetful- getting lost Lack of concentration, issues with concentration levels Regression in writing
Changes in personality/ behavior	Much crying Severe fatigue, nocturnal awakenings	Behavioral disorders Obstinate Peeing pants in school Personality changes, impulsive behavior, issues with sleep, loss of interest in activities that […] used to be interested in
Vision issues	Strabismus, nystagmus Sudden squint Went cross-eyed overnight	

One respondent is not accounted for in onset type breakout due to reportedly being a borderline late infantile/juvenile case

#### Constellation of symptoms

**Fig. 1:**
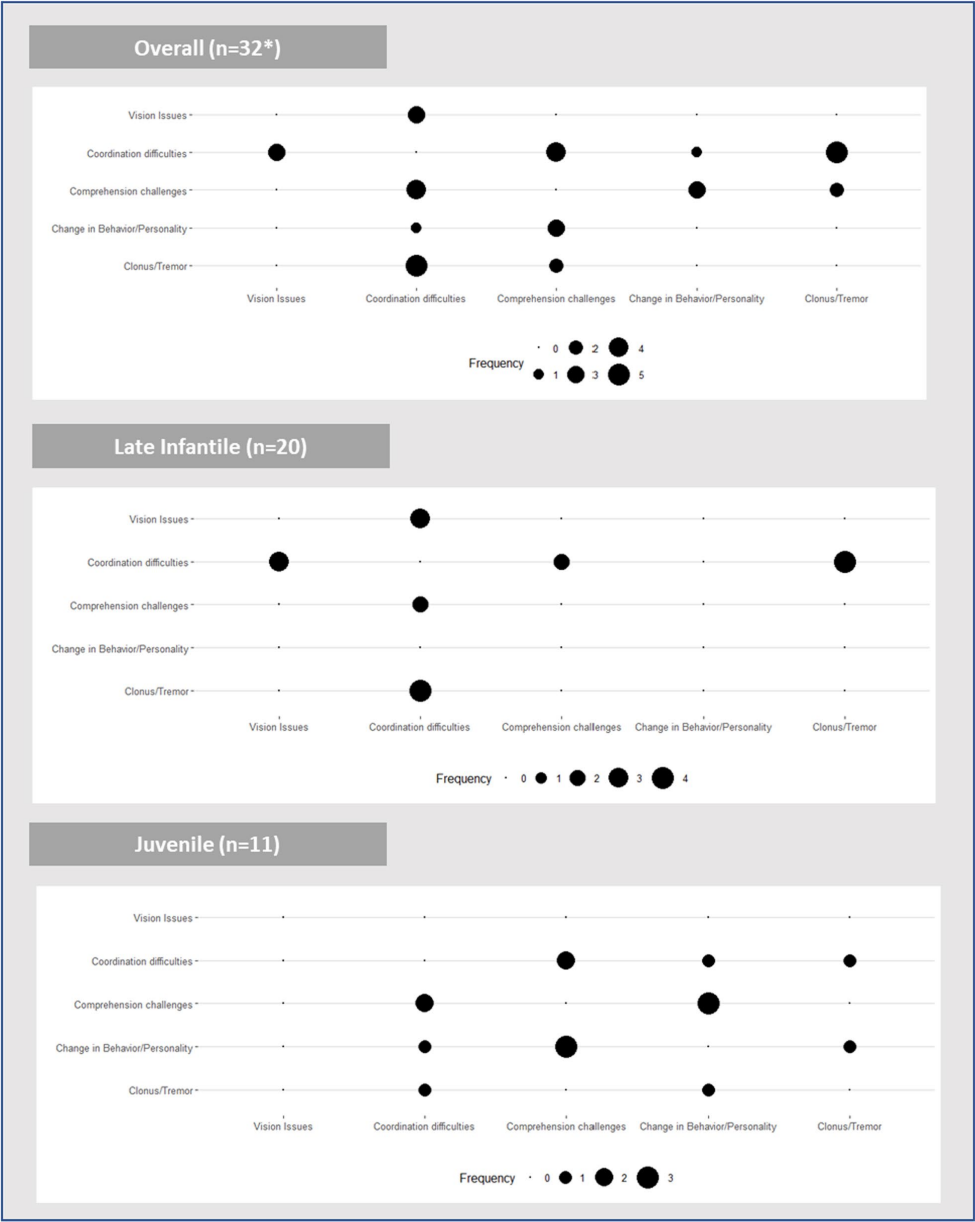
Constellations of caregiver-reported initial symptoms by onset type

In silo, these signs and symptoms described by caregivers could be characterized as non-specific and difficult to immediately connect to MLD. However, many caregivers reported a combination of symptoms that ultimately created a clear picture leading to a diagnostic workup for MLD (see Fig. 1).

#### Absence of causation for early signs of MLD

Caregivers reported an absence of perinatal injury prior to signs of MLD emerging. One caregiver explains, “when she was born, we had a great pregnancy. She was born healthy and she developed normally until 15 months”. None of the caregivers had unsolicited reports of pregnancy issues. Caregivers recall little explanation for the onset of symptoms that had first alarmed them. Those who reported developmental delays, stagnation, or regression (25/32, 75.0%) explained how their child was meeting milestones before noticing a disease-related stagnation in their milestones.

### Themes in early signs and symptoms described by caregivers

#### Most common early signs of MLD

Based on the coding and then grouping of caregiver-reported initial signs of MLD, coordination difficulties (75.0%), clonus/tremors (28.1%), and comprehension challenges (28.1%) were the most common types of initial symptoms described by caregivers. Physical symptoms, most commonly seen as coordination difficulties were characterized by specific signs described by caregivers such as gait spasticity and frequent falls. Cognitive symptoms, most frequently reported in the form of comprehension challenges, were often described by caregivers as signs such as appearing dazed and forgetful.

#### Development signs

More broadly, themes of developmental delays, stagnation, and regression are described by caregivers as the first indicators of concern. Seven of 32 MLD individuals reported signs of developmental delays (21.9%)—typically referring to their child not reaching certain expected milestones at typical pace, such as their child being “slower than the average child” or a “late walker”. Similarly, 21.9% of caregivers recall signs of developmental stagnation (7/32, 21.9%), which were described as “never being able to say more than a few words” or “never able to walk independently”. In some cases (11/32, 34.4%), caregivers’ first point of concern was when their child’s development began to regress. In these situations, signs of MLD were not flagged until the child had begun to lose some of their previously developed capabilities (e.g., losing ability to write).

#### Constellation of symptoms

Eighteen of 32 MLD individuals (56.3%) were reported with a constellation of symptoms that had developed in the initial presentation of the disease. The most common combination of symptoms reported was coordination difficulties plus clonus/tremor (5/32, 15.6%), and coordination difficulties plus comprehension challenges (4/32, 12.5%).

#### Differences in initial symptoms by age of onset

Nineteen of 20 (95.0%) with late infantile MLD were reported with physical symptoms as part of the early signs of their MLD. Similarly, caregivers noticed early physical symptoms in majority of juvenile individuals (8/11, 72.7%). Early cognitive symptoms were reported in 63.6% (7/11) of juvenile individuals versus only 10.0% (2/20) of late infantile individuals. Most common initial symptoms for individuals with late infantile onset MLD were coordination difficulties (18/20, 90.0%) and clonus/tremors (5/20, 25.0%). In individuals with juvenile onset MLD, coordination difficulties (6/11, 54.5%), comprehension issues (6/11, 54.5%), and changes in personality/behavior (5/11, 45.5%) were most frequently reported. Many of these early signs described in late infantile MLD can be classified as developmental delays (5/20, 25.0%) or developmental stagnations (5/20, 25.0%) while in the juvenile cases, caregivers tended to describe early symptoms as developmental regression (6/11, 54.5%) (Table 5). Coordination difficulties plus clonus/tremor were the most common symptoms reported in combination for those diagnosed with late infantile MLD (4/20, 20%). For those with juvenile MLD, caregivers most often recalled changes in personality/behavior and comprehension challenges happening together early on for their child (3/11, 27.3%) (Fig. 1).

**Table 5:** Caregiver-reported initial symptoms of MLD individuals by onset type

Initial symptoms (ordered by total)	Overall (n = 32*)	Late infantile (n = 20)	Juvenile (n = 11)
By early signs			
Coordination difficulties, n (%)	24 (75.0)	18 (90.0)	6 (54.5)
Clonus/Tremor, n (%)	9 (28.1)	5 (25.0)	3 (27.3)
Comprehension challenges, n (%)	9 (28.1)	2 (10.0)	6 (54.5)
Change in Behavior/Personality, n (%)	6 (18.8)	1 (5.0)	5 (45.5)
Vision Issues, n (%)	3 (9.4)	3 (15.0)	0 (0.0)
By symptom type			
Physical symptoms *(coordination difficulties, clonus/tremor, vision issues)*	28 (87.5)	19 (95.0)	8 (72.7)
Cognitive symptoms *(comprehension challenges, change in behavior/personality)*	10 (31.3)	2 (10.0)	7 (63.6)
By developmental signs			
Development delays	7 (21.9)	5 (25.0)	1 (9.1)
Developmental regression	11 (34.4)	5 (25.0)	6 (54.5)
Developmental stagnation	7 (21.9)	6 (30.0)	1 (9.1)

Initial symptoms were grouped into buckets based on similarities in response. There is overlap amongst respondents within each symptom group as many respondents listed more than one initial symptom

*One respondent is not accounted for in onset type breakout due to reportedly being a borderline late infantile/juvenile case

### Seeking medical consult after first clues

#### Symptoms triggering medical consult

When caregivers were asked to share what symptoms ultimately made them seek out medical advice, gait-related symptoms—including spasticity, inability to walk, slow to walk, or regressing ability to walk—were often reported as the main triggers to consult. Over the course of the diagnostic process, caregivers reported their children presenting with a constellation of symptoms. Both caregivers and clinicians struggled to put the different signs together, often prolonging the diagnostic journey. As one caregiver recounted, “nobody was putting the jigsaw pieces together and seeing the big picture”. Normal pregnancies or absence of birth complications often confounded timely diagnoses, as it was difficult to assess significance of subtle early signs without the ability to attribute to a specific incident.

#### First medical consult

**Fig. 2:**
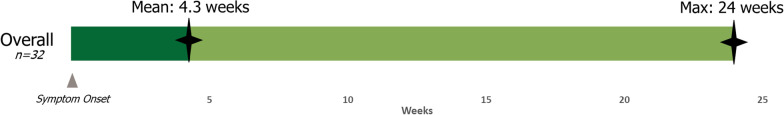
Time from symptom onset to first medical consult

After noticing the first concerning clues, 81.3% (26/32) of caregivers sought medical advice—with similar proportions for both late infantile and juvenile onset (80% [16/20] and 82% [9/11] respectively). Based on caregiver reports, the mean time to seeking medical consult was 4.3 weeks (range within 1–24 weeks) (Fig. 2). Time to seek medical consult was similar across onset types, averaging at 4.1 weeks (range within 1–16 weeks) for late infantile and 4.6 weeks (range within 1–24 weeks) for juvenile individuals. Generally, caregivers first visited a pediatrician, family primary care physician (PCP), general practitioner (GP) or (if in the UK) had an in-home health visit, before seeing a specialist. Those that did not immediately seek out medical advice (18.8%; 6/32) had waited due to lack of recognition of subtle, gradual symptoms, or misattribution to other confounding factors.

#### Reasons for not seeking immediate medical advice at first clues

One respondent’s child with late infantile MLD presented with frequent crying, which was difficult to interpret in a 15-month old child. Once they realized that their son had missed the milestone of walking, this prompted them to visit a clinician—this process took around 4 months.

In a different case of a juvenile onset individual, the caregiver clarified that medical advice was not immediately sought as they assumed that the behavioral issues were a sign of puberty. Once gait abnormalities presented, they decided to see a specialist. The time from presentation of behavioral symptoms to specialist visit was approximately 24 weeks.

In a third instance of a juvenile onset individual, the caregiver did not recognize their child's stroke-like arm movement; however, an occupational therapist at the child’s school observed the movements, which led to a medical consultation.

In another example, the caregiver did not seek out medical consult in their juvenile onset child until there was an evident accumulation, or constellation of symptoms—over 8 weeks, the MLD individual showed signs of coordination issues, severe fatigue, and nocturnal awakenings leading to medical consult.

### Time to diagnosis

#### Differences in time to diagnosis by onset type

Symptom onset for late infantile individuals were reported, on average, at 1.4 years old (range 0.3–2.5 years). Juvenile individuals were reported to have symptom onset, on average, at 7.2 years old (range 3.5–14.0 years); of these, early juvenile individuals reported symptom onset at 5.2 years old (n = 8) and late juvenile individuals reported first symptoms at 12.4 years old (n = 3).

Time from caregiver-reported symptom onset to the point of MLD diagnosis was variable, overall averaging 13.7 months. Mean time to diagnosis in late infantile individuals was 10.7 months (range 2.0–28.0 months), and 11.6 months (range 3.0–36.0 months) for juvenile individuals (Table 2). The time to diagnosis was much longer for one unique borderline late infantile/juvenile case which took 90 months to reach a diagnosis from when initial signs were observed (this case was not included in the breakout of time to diagnosis by onset type) (see Additional file 1: “Caregiver-Reported Borderline Late Infantile/Juvenile Patient Case” for more information). These analyses do not take into account potential differences in access to medical care between the countries where these families resided, which may have an influence on the diagnostic journey (Table 6).

**Table 6:** Time to diagnosis

Characteristics	Overall (n = 32*)	Late infantile (n = 20)	Juvenile (n = 11)
Age at first symptom onset (years)			
Mean	3.4	1.4	7.2
Median	1.5	1.5	5.6
Range	0.3–14.0	0.3–2.5	3.5–14.0
Age at diagnosis (years)			
Mean	4.4	2.3	7.8
Median	2.7	2.3	6.2
Range	1.2–14.3	1.2–3.5	4.5–14.3
Time between symptom onset and diagnosis (months)**			
Mean	13.3	10.7	11.6
Median	10.0	10.0	6.0
Range	2.0–90.0	2.0–28.0	2.0–36.0

*One respondent is not accounted for in onset type breakout due to reportedly being a borderline late infantile/juvenile case

**Excludes 2 individuals (6.3%) who were diagnosed through genetic testing as a result of a sibling’s diagnosis and received diagnosed at or before onset of symptoms (1 DE and 1 FR individual)

### Rapid progression from first signs of symptoms

Rapid disease progression is exemplified by one caregiver who shared that their healthy child began experiencing trouble walking at 15 months. From the first signs of disease onset to diagnosis (9 months) their child had lost almost all motor function and within the following month had lost all remaining motor function. 46.7% of MLD individuals (14/30) were reported by caregivers to have signs of developmental regression within 6 months or less from onset of initial symptoms, which is often months before a diagnosis is confirmed (13.7 months on average from symptom onset to MLD diagnosis as mentioned above).

### Role of caregivers’ perceptions and advocacy

#### Caregiver vigilance

Upon presentation of symptoms, caregivers recalled the feelings of first concern for their child. In what may have appeared as subtle and general signs, caregivers described their immediate impressions when watching their child’s behaviors change or stagnate. One caregiver described it as their “radar went off”—these themes of parental intuitions and vigilance were often mentioned (see Additional file 1: “Caregiver Perceptions” for quote from a caregiver).

### Role of non-clinical observers

#### Non-clinical observers

Signs and symptoms may first be noted by those involved in childcare, such as teachers and school staff. In an example provided by a caregiver, a nursery teacher’s observations prompted a follow-up visit with the GP for a child with juvenile-onset MLD. The initial signs were described by the caregiver as “anomalies”, such as concentration issues or non-age-appropriate behavior, until their nursery teacher observed these symptoms and was able to recommend further clinical action (see Additional file 1: “Non-Clinical Observers” for quote from a caregiver).

## Discussion

### Importance of early diagnosis

In summary, this study highlights the detailed caregiver language used to describe “red flags” suggestive of the early clinical picture of MLD. This study provides detailed insights into the initial signs and symptoms of MLD in the words of the caregiver which might trigger suspicion of disease for health care providers. There are few studies that focus specifically on the language used by caregivers of children with MLD—a key information source and important clue for clinicians to address individuals’ symptoms in a timely manner [15–18]. These first signs and symptoms are often missed during the critical window for early therapeutic intervention. Across all MLD individuals in this study’s sample, time to diagnosis took an average of 13.7 months, which meant that in many cases individuals were too far progressed to be suitable candidates for interventional therapies, leaving palliative and supportive care as their only options. As the European Group for Blood and Marrow Transplantation Guidelines suggests, in individuals with MLD, HSCT is “recommended in presymptomatic individuals or while neuropsychologic function and independence in activities of daily living remain good.” [19]. This urgency to treat is exemplified by the short window of opportunity before rapid and devastating disease progression occurs. In Kehrer et al. [20] the time from first motor symptoms to loss of walking without support, the point after which individuals experienced rapid disease progression, was only 8 to 27 months for those with late infantile and juvenile onset MLD respectively. Our study’s findings also illuminate the detriments of the rapid disease progression in MLD—34.4% (11/32) of the MLD individuals were considered to have developmental regression at the same time the first symptoms were noticed. Similarly, close to half of individuals (14/30, 46.7%) were reported to have experienced developmental regression within 6 months or less from onset of initial symptoms.

There is reasonable opportunity for disease stabilization post-transplant if the individual is presymptomatic or at an early enough stage in their symptom progression particularly in the juvenile form of MLD. The studies conducted by Beschle et al. [11], Groeschel et al. [21], and van Rappard et al. [22] emphasize certain prognostic factors attributing to disease stability post-transplant. Baseline characteristics of those who underwent a successful transplant included: presymptomatic individuals, acceptable gross motor function levels, low MRI severity scores, among others [11, 21, 22]. Beschle’s recent study also explores the short-term effects in an HSCT cohort that did not meet these positive prognostic factors, leading to rapid progression of symptoms at an even faster rate than non-transplanted individuals [11]. In our sample, only 5 MLD individuals (15.6%) were not too far progressed at diagnosis to be able to receive transplant. This rapid progression further underscores the short window for therapeutic intervention which is often missed due to delays in diagnosis. Those who were eligible for transplant were either diagnosed through early genetic testing based on a sibling’s diagnosis (2/5, 40%) or had a slower progressing juvenile onset type that allowed for some passing of time to recognize the constellation of symptoms forming (3/5, 60%). Similarly, Fumagalli et al. demonstrates how treatment effects of HSC gene therapy were found to be durable and clinically relevant even in early-symptomatic early juvenile individuals who were treated prior to moving into the rapidly progressive phase of the disease [23]. The factors associated with an effective and durable response to gene therapy also highlight the need for early disease identification and intervention to give individuals the best chance for disease stabilization. Even for individuals who are eligible for HSCT, there is a clear need for more effective options given the variability in response. In a single institution cohort of 40 MLD individuals who had undergone HSCT, the estimated 6-year survival was 50% for late infantile and 59% for juvenile forms of MLD [12]. Since the late infantile and juvenile forms of MLD account for close to 80% of all MLD individuals, there is a clear underserved population with an urgent need for both earlier recognition and intervention as well as alternatives to HSCT [24].

### Leveraging caregiver language to improve recognition of early signs of MLD

While findings from this study do further validate the most common early signs and symptoms of MLD as reported in similar studies [3, 16, 18, 25], its most distinct contribution is the added color on the specific language used by caregivers to describe these symptoms. This language in combination with the common early physical and cognitive signs can prove a useful resource in recognizing the clues that should trigger a workup for MLD (Table 7). Ultimately, a stronger understanding of the themes in caregiver language and common clues for suspecting MLD could be used to create a more structured phenotype ontology-based approach to the diagnostic algorithm. Similarly, these findings suggest further analysis of electronic medical records (EMR) could be used to inform machine learning and EMR flagging [26]. Furthermore, identifying opportunities to integrate these findings in HCP trainings and disease awareness efforts and establishing the evidence from this study and others (Table 7) as a validated resource for diagnosing MLD could prove a useful next step in establishing familiarity with the holistic picture of early MLD signs and accelerating path to appropriate biochemical testing.

**Table 7:** MLD first signs and symptoms from previous studies and complementary caregiver language from our existing study

	Previous studies			Complementary findings from our caregiver-reported survey		
	Source	Data point	Sample size	Supporting results	Sample size	Supporting caregiver language
Late infantile	Kehrer et al. [25]	In late infantile patients, 91.0% exhibited only motor symptoms	35	In late infantile patients, 95.0% were observed with early signs of coordination difficulties, clonus/tremor, and/or vision changes	20	**Late infantile MLD caregiver language: early physical signs**
						Coordination difficulties Delayed walking, difficult walking, strange posture Delayed when walking, many falls, gait sluggish Early to crawl, but late to stand up and hold onto things Has never been able to walk freely, twisted foot while walking Never walked, left was weaker than her right side Not progressing with walking (started taking first steps but did not progress after) Problems with motor development Slow motor skills Stagnation of motor development Started to lose balance Struggled to run- uncoordinated, clumsy, started walking late as a baby Trouble walking Unstable sitting, walking Unsteady gait Wasn’t getting on the couch anymore Wasn’t walking Clonus/tremor Clonus Developed a small tremor in hands Shake really bad after naps, foot tremoring Slight tremor Slight tremor, eye lid twitching (that pediatrician noticed on regular checkup) Tremors Very mid absences Changes in vision Strabismus, nystagmus Sudden squint Went cross-eyed overnight
	Kehrer et al. [25]	In late infantile patients, 9.0% exhibited motor and cognitive symptoms	35	In late infantile patients, 10.0% were observed with both physical and cognitive/behavioral early symptoms	20	**Late infantile MLD caregiver language: early physical + cognitive/behavioral signs**
						Slower than average child, wasn't walking, only 6 words, regressing Early to crawl, but late to stand up and holding on to things, gaps between achieving milestones was getting bigger
	Fumagalli et al. [3]	95.0% of late infantile subjects had a GMFC-MLD score > 1 (inability to walk independently) at 36 months	22	25.0% of late infantile patients were observed with development delays	20	**Late infantile MLD caregiver language: developmental delays in walking**
						Delayed walking, difficult walking, strange posture Delayed walking, many falls, gait sluggishness Early to crawl, but late to stand up and holding on to things Late walker, walking was stiff, often fell Slower than average child, wasn't walking
	Harrington et al. [16]	75.0% of late infantile patients experienced problems with gross motor function as initial symptom	16	90.0% of late infantile patients were reported with early signs of coordination difficulties	20	**Late infantile MLD caregiver language: gross motor function**
						Delayed walking, difficult walking, strange posture Delayed when walking, many falls, gait sluggish Early to crawl, but late to stand up and hold onto things Has never been able to walk freely, twisted foot while walking Never walked, left was weaker than her right side Not progressing with walking (started taking first steps but did not progress after) Problems with motor development Slow motor skills Stagnation of motor development Started to lose balance Struggled to run- uncoordinated, clumsy, started walking late as a baby Trouble walking Unstable sitting, walking Unsteady gait Wasn’t getting on the couch anymore Wasn’t walking
	Harrington et al. [16]	68.8% of patients in the late infantile group never learned to walk independently	16	30.0% of late infantile patients were reported to have development stagnation	20	**Late infantile MLD caregiver language: developmental stagnation**
						Could never walk independently Development not progressing Has never been able to walk freely Not progressing with walking (started talking first steps but did not progress after) She never walked Stagnation of motor development
	Harrington et al. [16]	62.5% of late infantile patients presented with fine motor or related symptoms (i.e., eye movement, eating or swallowing and hand tremors) pre-diagnosis	16	25.0% of late infantile patients were reported to have clonus/tremors 15.0% of late infantile patients were reported to have vision changes	20	**Late infantile MLD caregiver language: early fine motor signs**
						Clonus/tremors Clonus Developed a small tremor in hands Shake really bad after naps, foot tremoring Slight tremor Slight tremor, eye lid twitching (that pediatrician noticed on regular checkup) Tremors Very mid absences Changes in vision Strabismus, nystagmus Sudden squint Went cross-eyed overnight
	Beerepoot et al. [18]	The development of strabismus either clearly before, simultaneously with or shortly after gross motor symptom onset was reported exclusively in patients with a late infantile MLD form (27.0%, 17/63)	63	15.0% of late infantile patients were reported to have vision issues. All cases were reported in combination with coordination difficulties	20	**Late infantile MLD caregiver language: vision + gross motor early signs**
						Strabismus, nystagmus, slow motor skills Unsteady gait, sudden squint (18 months) Went cross-eyed over night and started to lose her balance
Juvenile	Kehrer et al. [25]	In early-juvenile patients, 61.0% exhibited only motor symptoms	18	In juvenile patients, 72.7% were observed with early signs of coordination difficulties, clonus/tremor, and/or vision changes	11	**Juvenile MLD caregiver language: early physical signs**
						Coordination difficulties Abnormal gait Broad-based gait Loss of balance, tripping Pain when walking, motor problems indicated by the teacher Unstable walking, never able to ride a bike Clonus/tremor Arm movement as if after a stroke Hand tremors
	Kehrer et al. [25]	In early-juvenile patients, 39.0% exhibited motor and cognitive symptoms	18	In juvenile patients, 18.2% were observed with both physical and cognitive/behavioral early symptoms	11	**Juvenile MLD caregiver language: early physical + cognitive/behavioral signs**
						Ability to do math, top of his class in 1st grade, 2nd grade couldn't monitor progress and didn't know he was struggling, 3rd grade couldn't add, struggled to run- uncoordinated, clumsy, started walking late as a baby Difficulty learning (learning vocabulary), abnormal gait, obstinate Lack of concentration, issues with concentration levels, very mild absences, little bit of regression (not age-appropriate behavior) Loss of balance, tripping, severe fatigue, nighttime awakenings
	Harrington et al. [16]	For juvenile patients, 56.3% had first symptoms related to changes in cognitive function	16	54.5% of juvenile patients reported initial comprehension challenges	11	**Juvenile MLD caregiver language: early signs of comprehension challenges**
						Ability to do math, top of his class in 1st grade, 2nd grade couldn't monitor progress and didn't know he was struggling, 3rd grade couldn't add Difficulty learning (learning vocabulary) Forgetful- getting lost Lack of concentration, issues with concentration levels Regression in writing
	Harrington et al. [16]	43.8% of juvenile patients had first symptoms related to social/behavioral function	16	45.5% of juvenile patients were reported with changes in behavior/ personality	11	**Juvenile MLD caregiver language: early signs of behavioral/personality changes**
						Behavioral disorders Obstinate Peeing pants in school Personality changes, impulsive behavior, issues with sleep, loss of interest in activities that […] used to be interested in
	Harrington et al. [16]	By the time of diagnosis, 56.3% of the patients with juvenile MLD had also experienced some decline in gross motor function	16	54.5% of juvenile patients were reported with developmental regression as an initial symptom	11	**Juvenile MLD caregiver language: developmental regression**
						Ability to do math, top of his class in 1st grade, 2nd grade couldn't monitor progress and didn't know he was struggling, 3rd grade couldn't add, struggled to run- uncoordinated, clumsy, started walking late as a baby Forgetful- getting lost, peeing in pants at school Little bit of regression (not age-appropriate behavior) Personality changes, impulsive behavior, issues with sleep, loss of interest in activities that […] used to be interested in Loss of balance, tripping, severe fatigue, nighttime awakenings Regression in writing

### Describing developmental signs of MLD

One of the themes highlighted in the above table are the development signs of MLD. When referring to “first signs and symptoms”, caregivers of late infantile MLD individuals often report signs of development delays and stagnation—as in, slowing and stagnation of their child meeting milestones. This finding is supported by the Kehrer et al. [27] retrospective study on early symptoms of late infantile and juvenile MLD, which reports that about half of the late infantile individuals never learned to speak in complete sentences after having acquired one- and two-word sentences within the normal time range, indicating stagnation in language development (Table 7). Consequently, Kehrer et al. recommends further investigation if absence of acquisition of complete sentences after initial normal language acquisition is noted [27]. Development delays and stagnation are not well circumscribed symptoms, but rather indicate a lack of developmental progress or gaining milestones. Throughout this study, we see this theme of the “persistent toddler” as described by caregivers– part of the challenge here is to highlight the lack of developmental progress, with the history of previously normal development as raising a “red flag” and prompting further investigation. Further, the nature of developmental issues makes it challenging to pinpoint the exact onset of first signs and symptoms. The insidious nature here is worth noting—onset may be a period of concern that develops over time as the delays reveal themselves and persist rather than a discrete time point. This subtlety is further confounded by the lack of perinatal injury or other clear cause.

### Symptom constellation

Eichler et al. [15] underscores the complex nature of MLD and how initial symptomatology can vary between and within patient types [15]. Our caregiver reports highlight that it is often a constellation of symptoms, rather than an individual symptom, that ultimately leads to diagnosis. These manifestations are a key characteristic of white matter disorders that affect the connecting fibers and thus multiple functions [28]. In our study, 56.3% of MLD individuals (18/32) were recalled by their caregivers with a constellation of symptoms developing prior to diagnosis. Fumagalli et al. [3] also underlined the sequence of symptoms that develop at disease onset. For instance, in early juvenile individuals, they found that even those who first presented with isolated behavioral or cognitive impairment, motor symptoms occurred within the next few months [3] (Table 7). While a focus upon multiple symptoms rather than individual ones may unfortunately warrant passing of time as further symptoms present, it is an important characteristic of the disease to keep in mind when thinking about caregiver reports. A confounding factor is the variability and non-specific nature of these earliest signs and symptoms that can add to the challenge of connecting these constellations of symptoms to MLD specifically. Nonetheless, recognizing the common language used by caregivers to describe the early constellation of symptoms can be another step towards earlier diagnosis.

### Differences in late infantile versus juvenile caregiver observations

Distinctions in caregiver descriptions for children with late infantile versus juvenile MLD may be more dependent on where these children are on the curve of motor and intellectual developmental milestones at the time of disease progression rather than any fundamental differences in the pathology of the disease. Changes in personality or behavior, for instance, are more noticeable in an older child at that stage of development as opposed to an infant where these cognitive signs may be less apparent. In juvenile cases, clearer behavioral symptomology can be distinguished from their normal behaviors and performance, providing a more concrete picture in terms of identifying early signs of regression. Conversely in late infantile cases, the developmental issues often reported can lack specificity and be more difficult to translate into a definitive sign of MLD.

### Absence of notable prior history

Confounding diagnosis is the lack of predicated indications of concerning signs in these MLD individuals. Caregiver observations gain further importance in light of normal pregnancy and birth history, creating a discrepancy and lack of explanation for the initial presenting symptoms. The absence of notable prior history becomes an important clue to diagnosis and a vital part of the full picture in assessing the first signs and symptoms of MLD.

### Call to action

The findings supported by this research provide a clear call to action for clinicians across specialties to support broader awareness of MLD and the key caregiver descriptions to look out. It is important for the medical community to recognize caregiver-reported observations consistent with MLD to direct individuals to immediate appropriate testing [29, 30]. Understanding the language used can be educational for caregivers, clinicians, and patient advocacy communities to keep an open and understanding dialogue around this condition and its related early manifestations. As demonstrated in Table 7, the caregiver language can be suggestive of specific physical, cognitive, or behavioral signs of MLD that can aid in early diagnosis. Increasingly critical assessments of these early signs can facilitate more rapid referral to proper specialists and streamline the referral pathway [23]. There is a recognizable opportunity for success that comes from providers taking “ownership” of the case as well. A challenge in rare diseases is that due to their specialized nature referrals are often thought to be the necessary and limiting factor. However, “referral” does not exclude taking “ownership”—the non-specialized nurse, for example, can refer while also pushing the PCP to initiate brain imaging.

It is also possible that other non-clinical persons, such as teachers and school behavioral specialists, pick up on these signs and escalate concerns as necessary to parents or clinicians. Those involved in the social sphere of interactions with these families may be well suited to connect individuals with the medical community, should their awareness of the neurological signs and symptoms be improved.

While this study does support means by which to help clinicians observe and therefore diagnose MLD earlier, we acknowledge that MLD is a rare condition with symptoms that are difficult to identify. It is unlikely, even with concrete anecdotes and language to listen for, that every general practitioner will be able to discern these subtle signs and make the connection to a leukodystrophy. Ultimately, newborn screening (NBS) will be crucial to diagnosing MLD as early as possible. NBS has precedent in other lysosomal storage disorders, such as MPS-1 where windows of therapeutic intervention are also best when performed before significant disease progression [31]. The development of MLD treatments recently approved, and on the horizon, also further highlights the need for NBS to ensure early patient identification and optimal therapeutic benefit.

### Limitations

It is important to note the potential limitations of the data as they are presented here. The respondents participating in this study are restricted to members of a convenience sample, such that their experiences and perspectives may differ from those of the real-world population of interest. These respondents were also asked to use their recollection rather than medical records for open-ended questions. This may have resulted in variability, recall bias, and inaccuracy of certain data. Given the small sample size, geographical generalizability and comparison across counties can be limited. However, relevance to caregivers and clinicians as a whole is not expected to be limited. However, due to the rarity of the disease this study highlights the importance of the caregiver experience that will enhance the body of literature for MLD recognition and treatment.

## Conclusion

The findings from this study offer insight into caregivers’ experiences and observations that can be used to raise the index of suspicion to trigger earlier investigations into MLD, furthering the objective to eradicate this diagnostic odyssey in MLD. These key findings continue to highlight the known challenges of missing milestones and diagnosing developmental delay, but add additional insights by providing the direct language used by caregivers, an important first step in the path to diagnosis. However, it is important to acknowledge the variability and lack of specificity in symptoms that will remain a confounding factor in making the accurate and more immediate connection to MLD. Broadening disease awareness by capturing these detailed cases of caregiver language used to describe the early indicators of MLD is crucial to ensure rapid testing and diagnosis of diseases such as MLD that are more easily treatable in the early stages with better outcomes. In lieu of a newborn screen, broader and earlier genetic testing based on these early signs is most likely to speed the journey to diagnosis. The findings supported by this research provide a clear call to action for clinicians across specialties to drive quicker attention to the first caregiver-reported signs to promote early identification of MLD—in the hopes of earlier therapeutic intervention and improved outcomes.

## Supplementary Information

**Additional file 1.** Verbatims from qualitative interviews with study participants.

## Data Availability

The datasets generated and analyzed during this study are not publicly available due to individual patient privacy protections.
