# Supplementary material for: Understanding caregiver descriptions of initial signs and symptoms to improve diagnosis of metachromatic leukodystrophy
Source: Orphanet J Rare Dis. 2022 Oct 4;17:370. doi: 10.1186/s13023-022-02518-z (PMC9531467; doi:10.1186/s13023-022-02518-z)
Supplement: Supplementary file 1 — Additional file 1. Verbatims from qualitative interviews with study participants. [file 13023_2022_2518_MOESM1_ESM.pptx]

## Slide 1
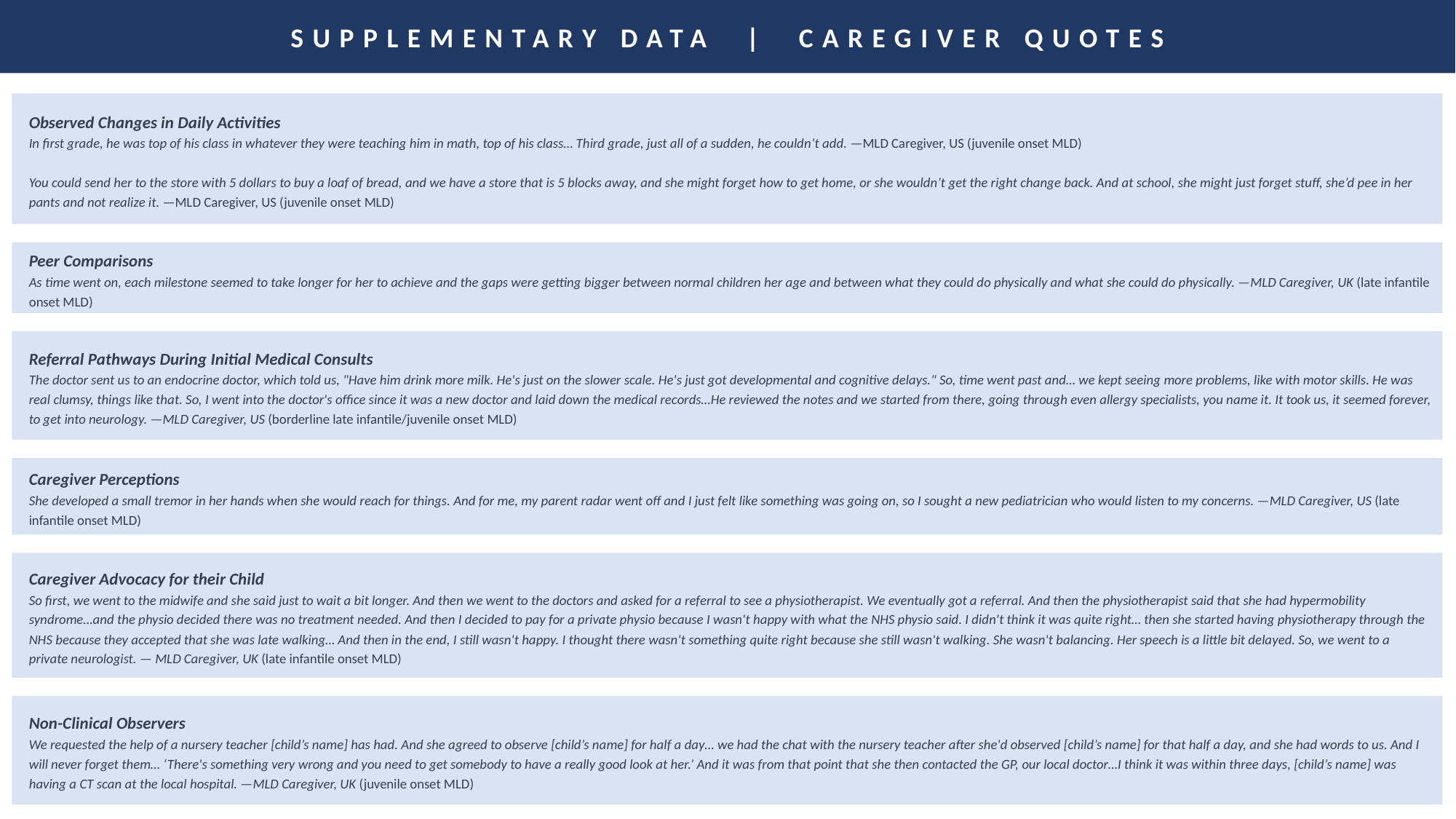

SUPPLEMENTARY DATA | CAREGIVER QUOTES
Observed Changes in Daily Activities
In first grade, he was top of his class in whatever they were teaching him in math, top of his class… Third grade, just all of a sudden, he couldn’t add. —MLD Caregiver, US (juvenile onset MLD)
You could send her to the store with 5 dollars to buy a loaf of bread, and we have a store that is 5 blocks away, and she might forget how to get home, or she wouldn’t get the right change back. And at school, she might just forget stuff, she’d pee in her pants and not realize it. —MLD Caregiver, US (juvenile onset MLD)
Peer Comparisons
As time went on, each milestone seemed to take longer for her to achieve and the gaps were getting bigger between normal children her age and between what they could do physically and what she could do physically. —MLD Caregiver, UK (late infantile onset MLD)
Referral Pathways During Initial Medical Consults
The doctor sent us to an endocrine doctor, which told us, "Have him drink more milk. He's just on the slower scale. He's just got developmental and cognitive delays." So, time went past and… we kept seeing more problems, like with motor skills. He was real clumsy, things like that. So, I went into the doctor's office since it was a new doctor and laid down the medical records…He reviewed the notes and we started from there, going through even allergy specialists, you name it. It took us, it seemed forever, to get into neurology. —MLD Caregiver, US (borderline late infantile/juvenile onset MLD)
Caregiver Perceptions
She developed a small tremor in her hands when she would reach for things. And for me, my parent radar went off and I just felt like something was going on, so I sought a new pediatrician who would listen to my concerns. —MLD Caregiver, US (late infantile onset MLD)
Caregiver Advocacy for their Child
So first, we went to the midwife and she said just to wait a bit longer. And then we went to the doctors and asked for a referral to see a physiotherapist. We eventually got a referral. And then the physiotherapist said that she had hypermobility syndrome…and the physio decided there was no treatment needed. And then I decided to pay for a private physio because I wasn't happy with what the NHS physio said. I didn't think it was quite right… then she started having physiotherapy through the NHS because they accepted that she was late walking… And then in the end, I still wasn't happy. I thought there wasn't something quite right because she still wasn't walking. She wasn't balancing. Her speech is a little bit delayed. So, we went to a private neurologist. — MLD Caregiver, UK (late infantile onset MLD)
Non-Clinical Observers
We requested the help of a nursery teacher [child’s name] has had. And she agreed to observe [child’s name] for half a day… we had the chat with the nursery teacher after she'd observed [child’s name] for that half a day, and she had words to us. And I will never forget them… ‘There's something very wrong and you need to get somebody to have a really good look at her.’ And it was from that point that she then contacted the GP, our local doctor…I think it was within three days, [child’s name] was having a CT scan at the local hospital. —MLD Caregiver, UK (juvenile onset MLD)
